# Determinants of nurse job dissatisfaction - findings from a cross-sectional survey analysis in the UK

**DOI:** 10.1186/s12912-020-00481-3

**Published:** 2020-09-18

**Authors:** Michaela Senek, Steven Robertson, Tony Ryan, Rachel King, Emily Wood, Bethany Taylor, Angela Tod

**Affiliations:** Division of Nursing & Midwifery, Department of Health Sciences, University of Sheffield, Sheffield, UK

**Keywords:** Nurse job satisfaction, Intention to leave, Staffing issues, Missed care, Leadership, UK

## Abstract

**Background:**

A lower recruitment and high turnover rate of registered nurses have resulted in a global shortage of nurses. In the UK, prior to the COVID-19 epidemic, nurses’ intention to leave rates were between 30 and 50% suggesting a high level of job dissatisfaction.

**Methods:**

In this study, we analysed data from a cross-sectional mixed-methods survey developed by the Royal College of Nursing and administered to the nursing workforce across all four UK nations, to explore the levels of dissatisfaction and demoralisation- one of the predictors of nurses’ intention to leave. We carried out logistic regression analysis on available data in order to determine what impacts job dissatisfaction.

**Results:**

In total, 1742 nurses responded to questions about working conditions on their last shift. We found that nearly two-thirds of respondents were *demoralised*. Nurses were five times more likely (OR 5.08, 95% CI: 3.82–6.60) to feel demoralised if they reported *missed care.* A perceived *lack of support* had nearly the same impact on the level of demoralisation (OR 4.8, 95% CI: 3.67–6.38). These findings were reflected in the qualitative findings where RNs reported how *staffing issues* and *failures in leadership*, left them feeling disempowered and demoralised.

**Conclusion:**

A large proportion of nurses reported feeling dissatisfied and demoralised. In order to reduce the negative impact of dissatisfaction and improve retention, more research needs to investigate the relationship dynamics within healthcare teams and how the burden experienced by RNs when unsupported by managers impacts on their ability to provide safe, good-quality care. These findings predate the current Covid-19 pandemic outbreak which may have had a further detrimental effect on job satisfaction in the UK and other nation’s nursing workforce.

## Background

The shortage of registered nurses (RNs) is a pressing issue across all four countries within the UK, with a similar trend and concern being observed across Europe and globally [1, 2]. In the UK, a drop in recruitment and retention of qualified nursing staff, as well as a rise in patient acuity, have been identified as main reasons for this workforce crisis [3]. The *Rising Pressure* report by the Health Foundation in 2017 showed that there was a 0.2% drop in the number of registered nurses, with a median leaver rate of around 15% in National Health Service (NHS) organisations [4]. Similarly, the Royal College of Nursing, UK, reported that from September 2017 to September 2018 there were 2532 more RN leavers than joiners in the nursing workforce. As a result, there were approximately 40,000 unfilled RN vacancies in 2019 [5]. Globally, the shortage of RNs was estimated to be 5.8 million [6].

Such shortages place health care systems under a burden during what might be termed ‘typical’ conditions. However, these systems come under extraordinary strain when hospital and community services are placed under ‘atypical’ conditions, such as those witnessed during the recent Covid-19 outbreak.

A review of systematic reviews of determinants of nurses’ intention to leave, found that the majority of included studies made a distinction between individual and organisational determinants of intention to leave. Individual determinants include age, gender, marital status, educational attainment, stress, burnout, commitment, job satisfaction, low serum cholesterol, weight and sleep disturbance [7]. Organisational determinants have centred on malfunctioning management and lack of supervision [8]. On an individual level, among all the multiple determinants of turnover in adult nursing, job dissatisfaction and nurse stress were some of the most important factors identified in the literature. For instance, individual studies by Larrabee et al., have shown that job dissatisfaction is predictive of both the intention to leave as well as actual turnover [9].

Whilst several studies have sought to address the range of predictors of intention to leave, some have furthered the field by use of a theoretical approach. One such attempt provides a link between job satisfaction and nurse turnover behaviour [10]. The theory categorised economic factors (pay, job market and training), structural factors (work environment, work context), and individual factors (psychological, demographic) as major determinants of nurses’ job satisfaction that influence behavioural intentions and turnover [11].

Further, a review by Coomper et al., that explored the components of job satisfaction and their impact on intention to leave, identified stress and leadership as main components that have the strongest impact on dissatisfaction among adult nurses and turnover. Whilst education and level of pay were found to be inconsistent, stress and leadership were identified as the best predictors of lack of satisfaction and intention to leave [10]. Previous literature has demonstrated the importance of leadership which is ethical and fair. Ozden et al. have raised the importance of fair leadership and leaders’ awareness of power-sharing and their effect on nurses’ job satisfaction in challenging times [12]. The findings showed that lack of fairness and ethical leadership can have particularly bad consequences on nurses during difficult times such as the COVID-19 pandemic [13].

Prevalence of job dissatisfaction among RNs was further highlighted through findings of a cross-sectional survey of 488 hospitals across Europe and the United States, which explored the level of dissatisfaction among RNs and associated outcomes. It found that job dissatisfaction was highest in Greece (56%), followed by Ireland (42%) and England (39%). Notably, a higher patient to nurse ratio (more than 10:1 in Greece and more than 8:1 for the latter two), as well as poor work environment, was reported in these countries [14].

A recent review of systematic reviews, exploring interventions to reduce adult nursing turnover, concluded that more high-quality primary research is needed to inform decision-making by human resource managers and organisations to improve retention strategies. The study included 9 systematic reviews in total. The review did not have definite findings due to the poor quality of evidence. Seven reviews were rated as moderate and two as being of poor quality. The main reasons for reviews being in the moderate rather than strong evidence category were the *lack of publication of a review protocol*, *unclear search strategy performed*, *the failure to have two reviewers check the selection and data extraction* and *not providing a list of both included and excluded primary studies.* More high-quality research would allow a better understanding the current main causes of RNs dissatisfaction based on primary research and is therefore pivotal to address this issue [15].

The aim of this study was to undertake a secondary analysis of a large UK wide data set in order to assess a set of self-reported individual and organisational predictors of nurses’ satisfaction/ dissatisfaction. We defined overall job satisfaction as a sum of all individual and organisational determinants and proposed to test a set of potential determinants, both individual and organisational, to see if and how they are associated with overall self-reported RN satisfaction. These data were collected during 2017, ahead of recent global pandemic conditions. Research about the determinants of dissatisfaction, as one of the predictors of nurses’ intention to leave is becomes even more relevant during a pandemic crisis such as COVID-19.

Whilst we recognise that there are many factors involved in nurses’ intention to leave, we have used the data available to us. In this study, we cannot predict ‘intention to leave’ but we are exploring job satisfaction as one of the previously known determinants of the intention to leave within a cohort of adult acute RNs.

## Methods

This study presents findings from a secondary analysis of an online-based cross-sectional survey of registered nurses from across the UK developed and administered by the RCN in May 2017 [16]. The RCN is the UK’s largest professional nursing body consisting of 450,000 members of registered and non-registered nursing and health care staff. A report from the survey, produced by the RCN and covering all questionnaire domains, is available on the RCN website [5].

We deployed an explanatory mixed methods study design. We began by descriptively exploring responses in the first part of the questionnaire. We then conducted a multivariate logistic regression modelling of the available data. From this, we initially developed a framework, and subsequently a thematic analysis, of the qualitative data. Finally, we (re)applied this to the quantitative data in a cyclical manner. The method therefore followed a process closely aligned with abductive reasoning. Definitions of abductive reasoning vary, however, all recognise it as a process where there is a cyclical and creative movement between the formulation of hypotheses and observed phenomena [17, 18]. While some identify the challenges of utilising abduction in qualitative studies [19], it is a form of reasoning well suited to mixed methods research as it develops claims supported from both deductive and inductively sourced evidence, in situations where the research is not driven exclusively by theory or by data [20].

### Study population

In May 2017, a staff survey of RN’s was carried out by the Royal College of Nursing (RCN). The survey was developed by the RCN, sent to all RCN members and was advertised on social media. The sample therefore consisted of both RCN members and non-members across the UK. The final sample responses comprised of 29,345 nurses. For the purpose of analysis, we identified from the data base and then included adult acute care nurses, which comprised 7040 RNs in total. In the UK, adult acute care covers all aspects of medical and surgical hospital in-patient care for those over 18 years of age but does not usually include in-patient mental health care. In order to provide a clear research focus on a specific group of RNs, we excluded RNs from the community, children’s nursing, mental health nursing and learning disabilities sectors. These settings will be analysed in subsequent pieces of work. The questionnaire did not ask the respondents to identify the specific hospital that they worked in for reasons of anonymity. It also did not ask for any demographic details. As a result, we were unable to carry out our analysis at the level of hospital and NHS trusts or consider the impact of demographics.

### Data sharing agreement and ethics

Before work commenced, a data sharing agreement was obtained between The University of Sheffield and the RCN. All data was anonymised prior to being shared with the research team. Ethical approval was obtained on 27/08/2019 from the University of Sheffield (Reference Number 026774) to conduct a secondary analysis of the anonymised RCN survey.

### Measured outcomes

We aimed to assess the determinants of the overall nurse dissatisfaction (with the job). In the survey, all responses were relating to RNs experience of their most recent shift.

The binary outcome of *Demoralised or Not Demoralised* was derived from response to the question: *I felt demoralised* (after my last shift). The RCN designed the survey and chose the phrasing of this question. The respondents could Agree, Strongly Agree, Disagree, Strongly Disagree with the statement. The Agree and Strongly Agree were merged as was Disagree and Strongly Disagree. We use the term *Demoralisation*, whilst recognising that *Dissatisfaction* is the more common variable used to predict *intention to leave* and *turnover*. However, respondents were in fact reporting on feeling *Demoralised,* which is a much stronger sentiment.

We aimed to test all independent variables available from the survey that have been identified in previous literature as determinants of nurse job dissatisfaction and demoralisation. The dependent variable was tested against all available independent variables in Table 1.

**Table 1:** Independent Outcome Variables from the Survey

Measured Outcomes	
Demoralised Y_N	I felt demoralised after my last shift
Understaffed Y_N	This was based on reported planned number of RNs for that shift
SupportY_N	I was provided with the appropriate support and supervision
MissedCareY_N	Due to the lack of time I had to leave necessary care undone
Action_RaisedConcernY_N	Was action taken after you raised concerns
OvertimeY_N	Did you work overtime
TakeBreakY_N	Were you able to take a break
Overtime Paid/Unpaid	If you worked overtime, was it paid
Agency Staff Ratio	Proportion of Agency RNs was calculated by dividing number of agency staff by total number of RNs on shift
Number of Patients	Self-reported number of patients that you cared for during your shift.
Sickness absence	Was there high absence due to sickness
Patient to Nurse Ratio	Number of RNs and number of patients during a shift

Respondents were also requested to respond to an open question asking them to describe and/or give examples of their last shift and any concerns or challenges they were able to observe. There was no word limit set on the length of replies to the open response question. These varied considerably in length and the amount of detail provided from a couple of sentences to 1–2 paragraphs. Due to the large amount of data from 1747 responses, 400 pages, we randomly sampled 10 pages of data for the analysis. This resulted in descriptive, narrative data from 368 individuals (20%) in total. While no formal test of data saturation was performed, a member of the team checked the emerging coding against a further 10 pages of data and no new codes were noted. In addition, Braun & Clarke suggest that for qualitative analysis of open question survey responses, a sample size of over 100 respondents is required for a large project [21]. We are therefore confident that with the responses from 368 individuals, and from checking an additional 10% of the data, that data saturation was achieved.

### Data analysis

An explanatory analysis was used to draw conclusions about the findings from the survey. In the first part of the analysis, we conducted univariate analysis with independent variables from the survey in order to assess the relationship and best model for predicting job dissatisfaction and demoralisation. A normality test showed that data was normally distributed for all responses. We then carried out a backwards logistic regression in order to select variables that best predict the model in order to determine which variables most affect nurses feeling Demoralised/Not Demoralised. We identified determinants that were most associated with affecting demoralisation.

From this point, a set of categories based on the findings from the regression modelling were used as an initial framework to organise and explore the qualitative data. Specifically, narratives around what led to dissatisfaction and feeling demoralised within this data set were considered. The qualitative data set was then further coded into sub themes and then overarching themes. Quirkos (v2.3) was used to assist in this phase of the analysis.

For the qualitative analysis, responses were analysed from 368 respondents who were representative of the overall respondents in terms of their clinical setting e.g. medicine, surgery. No analysis was performed to break down findings by these two clinical settings. Three researchers [SR, TR, MS] coded the responses.

The final stage of analysis was to more fully integrate the two data sets. Following processes of abduction [17], we aimed to understand the complexity of the interrelationships that exist between our data sets and the interpretations of them. We did this in order to integrate surface (semantic) and deep (latent) structures of a phenomenon; in this case understanding the determinants of job satisfaction. In practice, this process was achieved by research team meetings to iteratively align the quantitative and qualitative data sets and their analysis while simultaneously incorporating previous empirical work into these critical discussions. Again, in line with abduction, this allowed the juxtaposition of what is familiar with that not so familiar in order to generate robust explanations (that can be further tested) [17].

For reasons of flow and clarity, the results are presented as the two separate data sets and the more integrative analytical work is presented in the discussion.

## Results

Initially there were 7040 Adult Acute RNs in our sample. Out of those, 67.6% responded to the question on whether they *felt demoralised* (*N* = 4770), whilst the remaining 32.4% chose ‘Neither Agree or Disagree’ option, which was treated as ‘choose not to say’ and therefore as a missing value. Of the 4770 responses, 63.8% of RNs reported feeling *demoralised*, whilst 36.2% reported feeling *not demoralised* (see Table 2).

**Table 2:** Measured Outcomes Frequency

Measured Outcomes	Yes % (n)	No % (n)
Demoralised (Y_N)	60 (1048)	40 (699)
Understaffed Y_N	59.6 (1042)	40.4 (705)
SupportY_N	45 (787)	55 (960)
MissedCareY_N	51 (875)	49 (872)
Action_RaisedConcernY_N	60 (1041)	40 (706)
OvertimeY_N	72 (1261)	28 (486)
TakeBreakY_N	62 (1076)	38 (671)

To test the associations with nurses’ demoralisation we included the following determinants in the model:

In the binary logistic regression analysis, there were 3023 missing cases, or ‘neither agree or disagree’ options selected for at least one of the variables. These were treated as ‘no response/ ‘choose not to say’ due to the nature of the question and was therefore noted as ‘missing response’. The respondents who chose not to respond to this question could therefore not be included in the analysis. As a result, in total, 1747 valid cases were in the final analysis.

The overall model was statistically significant x^2^ (6) = 959,519, *p* < 0.001, predicting 82.7% of all cases.

Missed care (*p* < 0.001), lack of adequate support and supervision (*p* < 0.001), understaffed shift (*p* = 0.001), inability to take a break (p < 0.001), worked overtime (*p* < 0.001), action taken when concerns were raised (*p* < 0.001), were all significantly related to demoralisation.

Respondents who reported missed care, that is having to leave *necessary care undone*, were five times more likely to report being *Demoralised* (OR [5.02] 95% CI:3.67, 6.38). The RNs were 4.8 times more likely to be demoralised if there was a *lack of support* (OR [4.8], 95% CI: 3.67, 6.38). Other factors that were significantly associated were; whether *action was taken after they raised concern*, if they could not *take a break*, if they had to *work overtime* and if the shift was *understaffed* (see Table 3). Perceived high absence/sickness, percentage of temporary staff on the shift and number of patients seen, were excluded from the model.

**Table 3:** Predictors of adult acute nurse job demoralisation after a shift in a multivariable logistic regression model

Measured Outcomes	Adjusted OR	95% C.I.	Sig. *p* value
MissedCareY_N	5.021	3.82–6.60	*p* < .001
SupportY_N	4.840	3.67–6.38	p < .001
Action_RaisedConcernY_N	2.760	2.11, −3.62	p < .001
TakeBreakY_N	2.003	1.50–2.67	p < .001
Understaffed Y_N	1.914	1.45–2.52	p < .001
OvertimeY_N	1.812	1.33–2.47	p < .001

### Qualitative findings

**Fig. 1:**
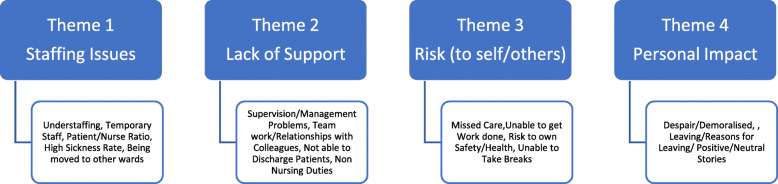
Themes

Responses were analysed from 368 respondents who were representative of the overall respondents in terms of their clinical setting e.g. medicine, surgery. No analysis was performed to break down findings by these two clinical settings. Three researchers [SR, TR, MS] coded the responses and those codes were subsequently grouped into 16 sub-themes. Further analysis revealed four main themes; *Staffing Issues*, *Lack of Support*, *Risk*, and *Personal Impact* (see Fig. 1). We describe each of the four themes below, using extracts from the data set to illuminate and confirm theme meanings. While each of these themes were reasonably equally weighted in terms of the number of responses made, as we will show, the first three seem to act synergistically to produce the depth of feeling expressed in the fourth theme. It is important to note that the analysis here, like the quantitative analysis, focused on dissatisfaction and demoralisation. Positive data, particularly relating to the first two themes, was also present and likely reflects the experiences of those 36% of RN’s who did not report feeing demoralised.

### Staffing issues

All respondents discussed staffing issues in their daily work and the challenges faced as a result. They spoke about a lack of adequate staff numbers, which resulted in higher than manageable patient to nurse ratios, and of the detrimental impact of this for both patients and staff:

*‘We are chronically understaffed, and I feel this regularly has a negative impact on patient care and staff wellbeing. We have patients that deteriorate quickly as well as a number of confused and high falls risk patients. I believe the lack of staff contributes to not being able to provide the highest level of care.’*

An aspect of this theme is the reference to personal, physical and emotional consequences of maintaining one’s professional role under such conditions and how these impact upon the quality of care provided:

*‘All staff are human and can only take so much of doing more than their workload. People end up being off sick due to exhaustion and stress. It's an impossible situation. Bottom line is understaffing to save money is as good as a chocolate tea pot. Eventually the staff left will melt and we'll be left with nothing but a mess.’*

Respondents described challenges which resulted from rota gaps as well as instances where, if they did have a full complement of staff, staffing resources would then be moved to cover gaps in other areas of the hospital. They described attempts to fill such rota gaps with temporary agency and bank staff. This posed a separate set of challenges due to the temporary nurse’s lack of familiarity with the ward and the patients:

*‘I have to work with a different nurse every shift. It's stressful trying to supervise bank or agency nurses as well as doing my own work. They aren't allowed to use certain pieces of equipment such as blood sugar monitoring or infusion pumps.’*

This theme demonstrates the challenging situations faced by RNs in their daily work. It demonstrates the complexity of this issue that leads to feelings of frustration and despair due to a lack of staff who are adequately trained and familiar with the ward setting.

### Failures in leadership and Organisational support

This theme describes the negative feelings attributed to a lack of support from hospital management, as experienced by respondents. This lack of support was experienced in a range of ways from simple disregard to being made to feel incompetent and even blamed for the poor state of patient care. The disregard encountered by RN’s was not only for themselves but also, they felt, extended to a disregard for patient’s needs:

*‘I feel our patients are behind us, but I do feel that upper management are disengaged with patients & staff’s real concerns and issues.’*

This apparent disconnect and disregard led to struggles with respondents trying to secure necessary staff or develop systems to help cope with excess workload. Such efforts were often undermined by managers leaving respondents disempowered and dissatisfied:

*‘Our site manager is unable to help us and refuses to let us save beds the night before knowing that we have these patients coming in.’*

*‘I have seen a large number of staff leave due to the pressures of being understaffed and the ward manager not helping on the ward. Nurses feel quite negative about matrons as they are not seen to do anything about understaffing.’*

At its worst, this disregard of concerns, and struggle to get support in providing quality patient care, was reflected back on the respondents in ways that led to stress, blame and feelings of professional vulnerability:

*‘Staff are made to feel incompetent by management when things are not done. It gets put down to poor time management on the nurses’ part. Went home from that shift feeling sad for the patients, angry with the management, absolutely exhausted and dreading the next shift.’*

*‘I try to do everything, but nothing is achieved. And yet the threat of disciplinary action hangs over nurses if anything goes wrong. […] We don't challenge and yet we are being challenged by the government and hospital bosses.’*

What is being described in this theme are broken relationships between respondents in the clinical area and those senior managers they rely on to provide practical and emotional support in delivering safe and effective care. Rather than being heard by those with the ability to help, many respondents report a perceived lack of action, or worse, actions that challenge their commitment and leave them feeling intimidated and demoralised.

### Risk to self/others

The understaffing and lack of support noted above generate risks to both the respondents and the patients they care for. In this theme, respondents give detailed accounts of the ways in which necessary care is left undone and the risk this poses to patients as well as the risks that they face during their day-to-day work. Examples provided point to the immediate risks to patient well-being and to the subsequent impact on the wider health care system.

*‘Looking after 15 patients you cannot meet patients’ needs, results in cutting corners and not always delivering safe care. This results in extra pressures due to the bigger patient workload. Wound dressings are not being renewed when they should be which results in further infections and extra time in hospital.’*

*‘I often have to look after 10 patients and the medication round takes so long to administer, some patients have medication up to 2 hours late. I also cannot check observations as frequently as I would prefer.’*

As well as describing observations about the increased risk of adverse events to patients, RNs described risks to themselves. They noted that whilst they tried to care for their patients, their own health and safety was not prioritised and was therefore at risk. They often described putting themselves in situations where their own safety was being jeopardized:

*‘I received a needle stick injury at work. Patient who was needle phobic knocked needle into me. I was unable to follow correct procedures after injury. No staff cover for me to go to A&E straight away (I had to go 2 days later- waited 3hrs to be seen) to have procedural blood tests done.’*

Respondents noted the primary and secondary impact of not being afforded enough resources to carry out the care they aspire to. The primary impact is noted in relation to patient care, with a secondary consequence for themselves:

*‘I am feeling sick with stress and fear for patient safety.’*

The respondents spoke about being in situations where they had to make choices between their own safety, versus the safety of their patients. The situation described below is an example where highly vulnerable patients, and a vulnerable staff member, were left risk-exposed by chronic under-resourcing:

*‘When I left work there was no night nurse to hand over to for the 2 bays of patients I was looking after, should have been one bay and a side room. I'm 36 weeks pregnant in an acute respiratory ward. I stayed for half an hour to wait whilst they tried to find a cover nurse but sadly the whole hospital was in the same situation. I was told to go by the nurse in charge that shift. This left 2 nurses looking after 35 patients. 4 of which are high dependency on NIV. Not safe!’*

This theme demonstrates the awareness of a heightened risk of adverse outcomes when staffing resources are short. Respondents recounted not only awareness of times when they were failing to deliver adequate patient care but also of the times when they had to make choices between safe patient care and their own wellbeing – usually erring toward neglecting their own wellbeing and putting patients first. This compromise between the patients care and safety and their own wellbeing led to mental distress, anxiety and extreme feeling of dissatisfaction; in short, it had a significant personal impact for these nurses.

### Personal impact

The three themes described above point to a set of resource and organisational conditions that often resulted in the failure to deliver the best care. Collectively, these have huge personal impact on the respondents with concomitant consequences for their wellbeing and job satisfaction. Respondents described strong feelings of despair and being demoralised and highlighted how these feelings about their job impacted both their personal and their professional lives:

*‘Emotionally exhausted after shift, being in a bad mood to family, crying at home because of the pressures. Feeling physically unwell during shift as no time to rest/take break for air or drink of water. Depressing knowing that you won't leave work on time. Busy workdays are good and can make you feel energised and positive but being overstretched on every single shift and worrying about mistakes being made is exhausting.’*

They described scenarios which lead them to consider ways in which they can leave or change their professions. They also described scenarios which had led their colleagues to leave the profession:

*‘We stated that 2 registered nurses to look after 19 patients (11 of whom were post-operative) and 10 of whom needed discharging later in the day… I have been qualified for one year and I have already started the process of going into a different career. I will have given up nursing within the next 18 months.’*

The observations which the respondents describe suggest that the obstacles faced during work have a profound negative influence on their lives. The negative experiences during their working hours diffuses into their after-work hours impacting their personal and home life to an extent that they are struggling to deal with. The situations which form an overall experience leave them feeling a sense of despair and hopelessness; these then form the basis for their intentions to leave. The quote below draws together findings from the first three themes showing how they collectively build to create strong feelings of dissatisfaction and demoralisation that impact on personal life:

*‘Some days nursing affects my whole life. I'm tired, I'm demoralised and I'm stressed. I try my hardest to give my best to my patients, but every day is like spinning plates and it feels like if my concerns are raised to managers then I am to blame for not coping or managing my workload properly. It affects my family life as some days I wake up at 2am worrying about something work related. We have a high sickness rate, so we are down on staff and we have a high turnover of staff as people are always leaving.’*

Ultimately, as this quote and theme suggest, intentions to leave may become actions once the personal and professional situation becomes unmanageable and the losses become far more than benefits of doing a job that they describe as one that they once loved.

### Summary of findings

The results from the survey show that nurses were most likely to feel demoralised if they *missed care,* followed by if they reported *lack of support* and *Lack of action when concerns are raised*. Being *Unable to take a break, Understaffing*, and *Having to work overtime* were also significant factors.

Qualitative findings demonstrate significant concerns about inadequate staffing and how this leads to demoralisation and dissatisfaction when safe and effective care cannot be provided. These feeling are compounded by a lack of managerial support which can lead to feelings of stress, blame and professional vulnerability. Staff often have to make choices between risks to the patient and risk to their own wellbeing. In such situations nurses tend to prioritise patients. The inability to provide quality care, have concerns addressed by management, take breaks and finish on time take a personal toll on nurses leading them to consider leaving the job and the profession.

## Discussion

Results here provide a picture of the factors that generated demoralisation among nurses in the UK in the period leading up to the COVID-19 pandemic. A recent review of the experience of nurses during epidemics associated with respiratory conditions, revealed that the quality of leadership and organisational factors, as well as staffing resources, leading into such events has a significant impact upon how the health care system is able to perform [22]. With this in mind, our paper is useful in being in a position to describe the conditions many nurses were working under at the time of the Covid-19 pandemic onset, and from which we can begin to understand the healthcare system’s operational performance during these events.

While our quantitative data showed that *leaving necessary care undone* and *lack of support* were the factors most likely to impact on feeling demoralised (and therefore on job dissatisfaction), the qualitative data suggest a strong emphasis on adequate staffing. On closer consideration, it becomes apparent that it is not understaffing per se that is the main issue of concern but the consequences of this and the lack of support to avoid or prevent these consequences. The primary focus of the nurse is on the ability to provide safe and effective patient care and dissatisfaction and feeling demoralised occurs when this cannot be achieved and those in more senior positions do not respond to their expressed concerns. Given that missed care has been seen as the mediator linking lower registered nurse staffing levels with increased patient mortality [23], it seems no surprise that not being able to provide adequate care is one of the greatest predictors of job dissatisfaction among the respondents [24].

In light of our findings of the impact of missed care on dissatisfaction and feeling demoralised, any approach aimed at increasing RNs’ satisfaction and retention should focus on interventions which allow RN’s to provide safe and effective nursing care. In addition to evidence that understaffing increases the occurrence of missed care and therefore job satisfaction, Senek et al. have recently demonstrated that ensuring adequate staffing numbers by covering rota gaps only with temporary staff (agency and bank staff) does not necessarily lower the occurrence of missed care [25]. In recent years, a solution to severe understaffing has been to deploy temporary agency and bank RNs, who often rotate between specialities and hospital sites. This temporary deployment means that they are often not familiar with the setting, staff or patient groups they are working with. Not surprisingly then, it has been shown that there is more missed care on shifts that have higher proportions of temporary staff than on understaffed shifts [25]. Therefore, in order to ensure that RNs can achieve a satisfactory level of quality care provision, it is not only adequate staffing levels but also the right type of permanent staff, which allows for continuity of care and team building that can reduce the occurrence of missed care. As indicated by our findings, this has a significant impact on RN’s satisfaction, and the likelihood of RN’s remaining in the profession [10].

The importance of *not being able to take a break* (OR = 2.0) and *working overtime* (OR = 1.8) can also be accounted for in this way. The quotes provided mention these issues, but they are a secondary narrative to the primary concern of being able to provide adequate, safe and effective care. These also link into another important finding, that of the choices nurses are forced to make when staffing levels are low, support is lacking and patient care is therefore at risk. Missing breaks and working overtime are resorted to in order to ensure necessary patient care is not missed, or at least to minimise the amount that is missed. In this way, they represent a secondary, but still important, mechanism in generating job dissatisfaction by forcing nurses to choose between their own needs and those of their patients. This resonates with previous findings which showed that nurses’ inability to take breaks was due to *patient load*, *unpredictability of patient needs* and *reluctance to burden other nurses* [26].

Collectively, the inability to provide quality care, have concerns addressed by management, take breaks and finish on time take a personal toll on nurses. It impacts their physical and mental wellbeing. It leaves them feeling undervalued, disempowered, intimidated, and vulnerable to committing clinical errors and the professional and personal consequence of this. It affects their relationships outside of work and, ultimately, it leads them to consider whether to leave the job and even the profession. Similar findings have been reported where psychological disempowerment of RNs resulted in job dissatisfaction, lack of organisational trust and staff nurse burnout [27, 28]. Although the questionnaire did not specifically ask whether the respondent intends to leave the profession, these factors have previously been reported to contribute to RNs job dissatisfaction which is a predictor of intention to leave [7].

These findings have relevance for how managers and organisations may consider staffing and supporting nurses. Sellgren et al. have shown that nurses job satisfaction is lower when managers are ‘invisible’ whereas strong facilitative leadership behaviours create an environment that increases job satisfaction [29]. They further note that when managers lead with kindness and respect, and in ways that demonstrate ethical leadership [12, 13], it is more likely that staff also demonstrate the same behaviour towards the patients. Similarly, Morsiani et al. demonstrated that when managers adopted leadership styles focused on monitoring and intervening to correct errors it has negative impact on nurses’ levels of job satisfaction whereas transformational leadership styles that involve respect and care for others improve staff satisfaction [30]. It may also be worth managers taking a collective nursing team view on what constitutes sufficient numbers and mix of staff when planning the nursing roster. Adams and Bond showed that when staff considered there were sufficient numbers of skilled staff rostered and organized appropriately, nurses’ job satisfaction was greater [31]. Importantly, they also linked this to non-hierarchical leadership styles and management that was respectful and patient-centred [32].

We have reported on the level of RNs work morale before the COVID-19 pandemic. During and post pandemic it is predicted that strain and work-related stress are much greater, as reported in previous health emergencies. For instance, during and following the Severe Acute Respiratory Syndrome (SARS) outbreak, Taiwanese RNs reported high levels of stress, even more so in moderate-risk areas than those working in high-risk areas [33]. A cross-sectional survey reported that nearly 8% of the nurses thought they should not care for SARS patients and considered resignation, mainly due to increased work stress and perceived risk of fatality. These findings are important in view of the current COVID-19 pandemic and any future impending outbreaks [34]. Prior to the COVID-19 pandemic, UK RNs intention to leave rates were reported to be between 30 and 50%. The evidence-base from studies on SARS and Middle Eastern Respiratory Syndrome (MERS) epidemic outbreaks in South Korea [35] suggests that this phenomenon is exacerbated in a deadly disease outbreak. Currently, in the UK, concerns over safety, reported lack of personal protective equipment and high fatality rate of health care professionals will further increase work-related stress during the COVID-19 pandemic. The unprecedented crisis caused by the pandemic may therefore have a further negative impact on nurse retention.

### Limitations

The variables that were tested from the survey were mainly job-related, interpersonal, and organisational factors. The personal and individual factors such as age, gender and level of experience were not available to us and could therefore not be included in the analysis. Similarly, we cannot be sure that the qualitative data represents an accurate spread of views from across the adult nursing population.

### Future direction of research

It seems clear that further research is needed to address the root causes of RN’s dissatisfaction. Future work should investigate the relationship dynamics within healthcare teams and how the burden experienced by RNs when unsupported by managers impacts on their ability to provide safe and effective care. Our data shows that RNs feel that there is limited recognition of the wider issue of understaffing and, when the issue is raised, they are often intimidated into continuing to work under these difficult conditions. In this instance, we have demonstrated their issues with management, but we recognise that RNs are part of a health care team that consists of many different roles. Therefore, to address this wider issue, there needs to be involvement of the whole team and all stakeholders involved. However, these issues will forever remain if RNs are experiencing severe workloads and poor staffing levels that put their patients at risk due to missed care [36]. Understaffing is an underlying issue, which needs to be recognised. For this to be dealt with effectively, it is not enough to train more people to be nurses when the dissatisfaction and the leaving-rate is high for those who are currently in the profession.

## Conclusion

A high proportion of feeling demoralised and dissatisfied was reported by registered nurses and was most likely to occur as a result of missed care and lack of support and action when concerns were raised about this. Whilst some of these findings are consistent with those from previous studies, their level of impact and the mechanisms by which they cause a detrimental effect on nurses’ moral and job satisfaction have not previously been fully discussed. Addressing the issues highlighted here will be important in addressing the root causes of RN dissatisfaction and thereby improving retention and reducing the high turnover rate among nurses. We intend that this paper contributes to the national and international debate about how the profession is regarded by governments and organisations involved in healthcare, both during pandemic conditions and during periods of recovery.

## Data Availability

The survey data analysed in this study was not publicly available. The data that support the findings of this study are available from Royal College of Nursing, but restrictions apply to the availability of these data. To access the data, we obtained a Data Sharing Agreement and Ethical Approval for the current study.

## Abbreviations

NHS: National Health Service
RN: Registered Nurse
RCN: Royal College of Nursing
SARS: Severe Acute Respiratory Syndrome
MERS: Middle East Respiratory Syndrome
